# *MYC* amplifications in myeloma cell lines: correlation with MYC-inhibitor efficacy

**DOI:** 10.18632/oncotarget.4245

**Published:** 2015-06-02

**Authors:** Toril Holien, Kristine Misund, Oddrun Elise Olsen, Katarzyna Anna Baranowska, Glenn Buene, Magne Børset, Anders Waage, Anders Sundan

**Affiliations:** ^1^ K.G. Jebsen Center for Myeloma Research, Department of Cancer Research and Molecular Medicine, Norwegian University of Science and Technology, Trondheim, Norway; ^2^ Department of Immunology and Transfusion Medicine, St. Olav's University Hospital, Trondheim, Norway; ^3^ Department of Hematology, St. Olav's University Hospital, Trondheim, Norway; ^4^ CEMIR (Centre of Molecular Inflammation Research), Department of Cancer Research and Molecular Medicine, Norwegian University of Science and Technology, Trondheim, Norway

**Keywords:** oncology, carcinogenesis, molecular and cellular biology, signal transduction, cell cycle

## Abstract

In multiple myeloma, elevated MYC expression is related to disease initiation and progression. We found that in myeloma cell lines, *MYC* gene amplifications were common and correlated with *MYC* mRNA and protein. In primary cell samples *MYC* mRNA levels were also relatively high; however gene copy number alterations were uncommon. Elevated levels of MYC in primary myeloma cells have been reported to arise from complex genetic aberrations and are more common than previously thought. Thus, elevated MYC expression is achieved differently in myeloma cell lines and primary cells. Sensitivity of myeloma cell lines to the MYC inhibitor 10058-F4 correlated with MYC expression, supporting that the activity of 10058-F4 was through specific inhibition of MYC.

## INTRODUCTION

Multiple myeloma is the second most common hematological malignancy and accounts for about 2% of cancer-related deaths. The cancer cells arise from post-germinal center plasma cells and usually reside in the bone marrow. Myeloma is preceded by a benign condition termed monoclonal gammopathy of undetermined significance (MGUS), [1] and the annual risk of progression to myeloma is about 1%. [2] Treatment of multiple myeloma has improved during the last decades by the introduction of proteasome inhibitors such as bortezomib and carfilzomib as well as immunomodulatory drugs (IMIDs) such as thalidomide and lenalidomide. However; the median overall survival time from diagnosis is still no more than 5–7 years. [3, 4]

The transcription factor c-MYC (hereafter termed MYC) and the related N-MYC and L-MYC oncogenes are involved in the development of up to 70% of all cancers. [5] Under normal conditions MYC increases cell proliferation and halts differentiation. [6] Abnormal MYC activity has been shown to be associated with many features of cancer cells including cell metabolism and proliferation. [7] In multiple myeloma it was commonly thought that activation of MYC was a late-stage event. [8] The importance of MYC in myeloma disease progression has lately become clearer, and increased MYC activity has been implicated in progression from MGUS to full-blown myeloma. [9–12] Recently, two papers showed that complex rearrangements positioning MYC in the proximity of super-enhancers caused elevated MYC expression in primary myeloma cells. [13, 14] Moreover, MYC was shown to be the most frequent translocation partner in aberrations involving the immunoglobulin light chains. [15] Altogether, MYC rearrangements were found in nearly half of the myeloma patients leaving MYC the most commonly mutated gene in multiple myeloma. [13]

We have earlier shown that MYC expression was important for *in vitro* survival of myeloma cells using different approaches for targeting MYC. [16–18] The question we wanted to address in this study was whether the vulnerability of multiple myeloma cells for MYC inhibition correlated to cellular levels of MYC. Pharmacological targeting of MYC activity has been challenging. One option is to use small molecule inhibitors that target MYC-MAX heterodimerization thereby preventing transactivation of MYC target genes. [19, 20] We found that the small molecule inhibitor of MYC, 10058-F4, suppressed proliferation and survival of myeloma cells, arguing for a distinct role of MYC in multiple myeloma. The importance of MYC was further supported by an inverse correlation between IC50 of the inhibitor and the level of MYC in myeloma cell lines.

## RESULTS

**Figure 1:**
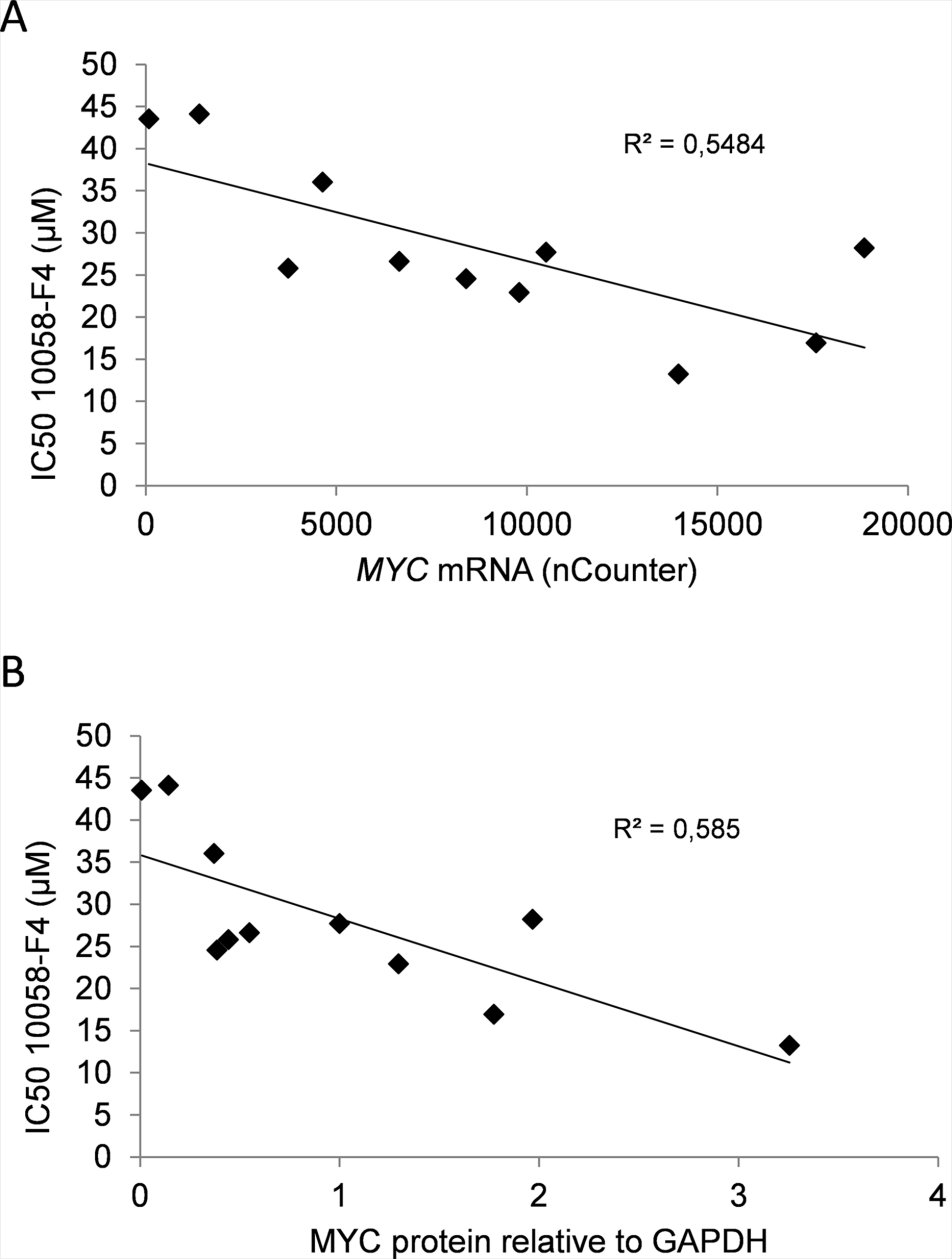
*MYC* gene copy numbers determine expression of MYC mRNA and protein in myeloma cell lines In a panel of myeloma cell lines the levels of *MYC* gene copy numbers as measured by PCR was related to **A.** *MYC* mRNA measured using nCounter, and **B.** MYC protein levels measured using immunoblotting and normalized to GAPDH. The slope and R^2^-values are shown in the plots.

We have earlier shown that the small molecule MYC inhibitor 10058-F4 induces apoptosis in myeloma cell lines and primary cells. [17, 20] The inhibitor downregulated MYC protein and mRNA in a dose-dependent manner in myeloma cells (Supplemenatry Figure 1A–1C). We wanted to find out if the baseline MYC expression could determine myeloma cell sensitivity to 10058-F4. A panel (*n* = 11) of myeloma cell lines were treated with increasing amounts of inhibitor for three days. The combined effects on cell proliferation and viability were determined using CellTiter Glo which measures the ATP content in cells (Supplementary Figure 2). IC50 values were determined from dose-response curves and related to *MYC* transcript numbers measured by the nCounter Nanostring technology (Figure 1A, Supplementary Figure 3A) and protein levels using immunoblotting (Figure 1B, Supplementary Figure 3B, 3C). There was a negative correlation between IC50 values and mRNA (R^2^ = 0, 548) or protein (R^2^ = 0, 585) levels. Taken together, the correlation between MYC expression and sensitivity to the 10058-F4 compound, supports that 10058-F4 is a relatively specific inhibitor of MYC activity. Secondly, the finding that the cell lines with the highest MYC concentration were the most sensitive suggests that cell lines expressing high levels of MYC are more dependent on the MYC expression for proliferation or survival than cell lines expressing lower amounts of MYC.

**Figure 2:**
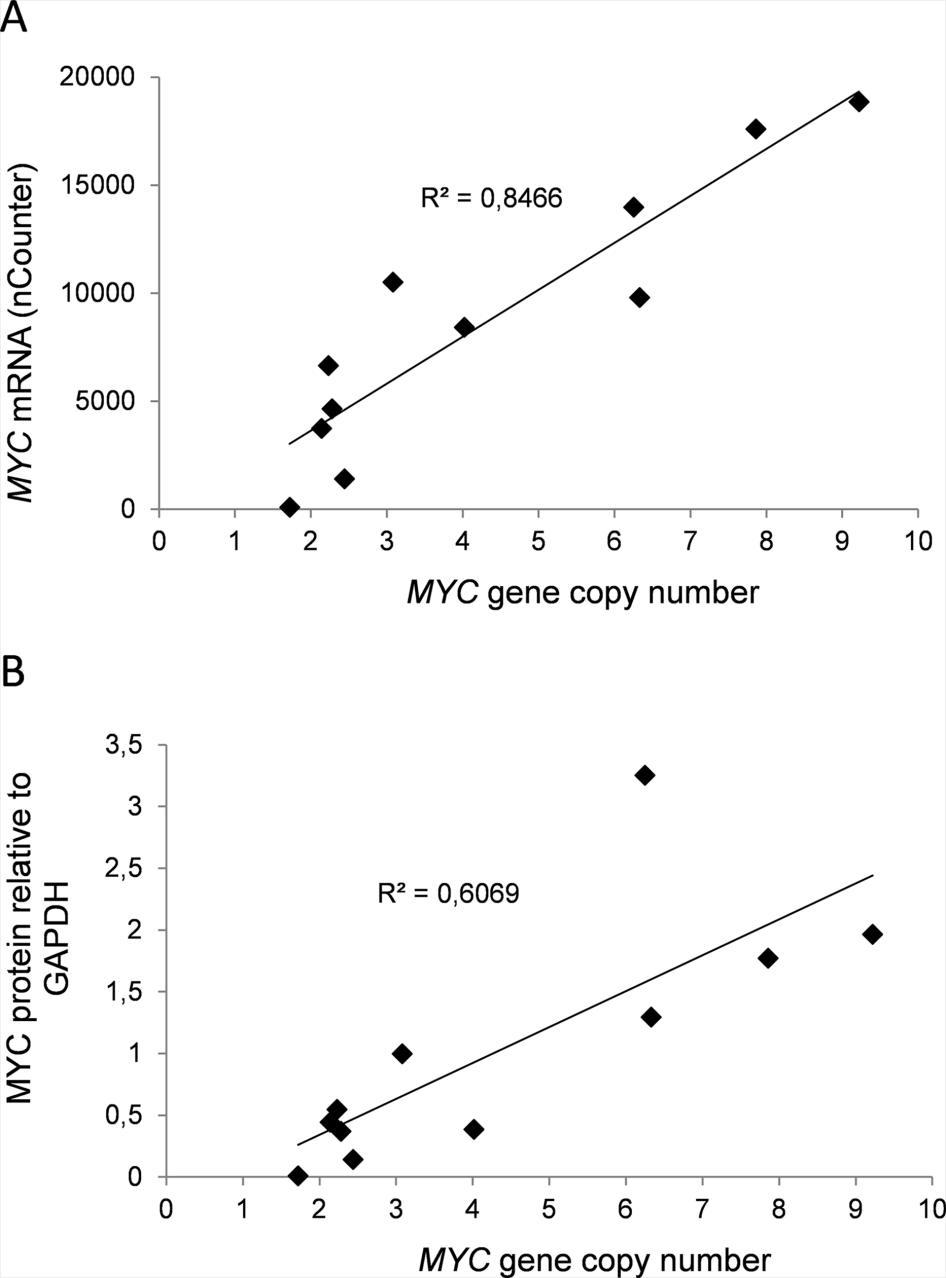
Expression of MYC in myeloma cell lines correlated positively with sensitivity to MYC inhibition The IC50-values of the MYC inhibitor 10058-F4 calculated from the results shown in Supplementary Figure 2 was compared with **A.** *MYC* mRNA values or **B.** MYC/GAPDH relative protein levels. The slope and R^2^-values are shown in the plots.

Next, we measured *MYC* gene copy numbers in all 11 myeloma cell lines using PCR with primers for exon 3 (Supplementary Figure 3D) and correlated the copy numbers with *MYC* mRNA, as well as with protein levels (Supplementary Figure 3A, 3B and 3C). In cell lines, the MYC gene copy numbers varied from two to nine. The measured copy numbers were almost identical using primers that were specific for exon 1 and exon 2 (Supplementary Figure 3D), indicating the presence of the whole gene rather than fragments of the gene. Interestingly, the *MYC* gene copy numbers correlated well with both mRNA (R^2^ = 0.847) and protein (R^2^ = 0.607) levels (Figure 2A and 2B). The results thus indicate that the main determinant of elevated MYC expression in myeloma cell lines is amplification of the *MYC* gene.

**Figure 3:**
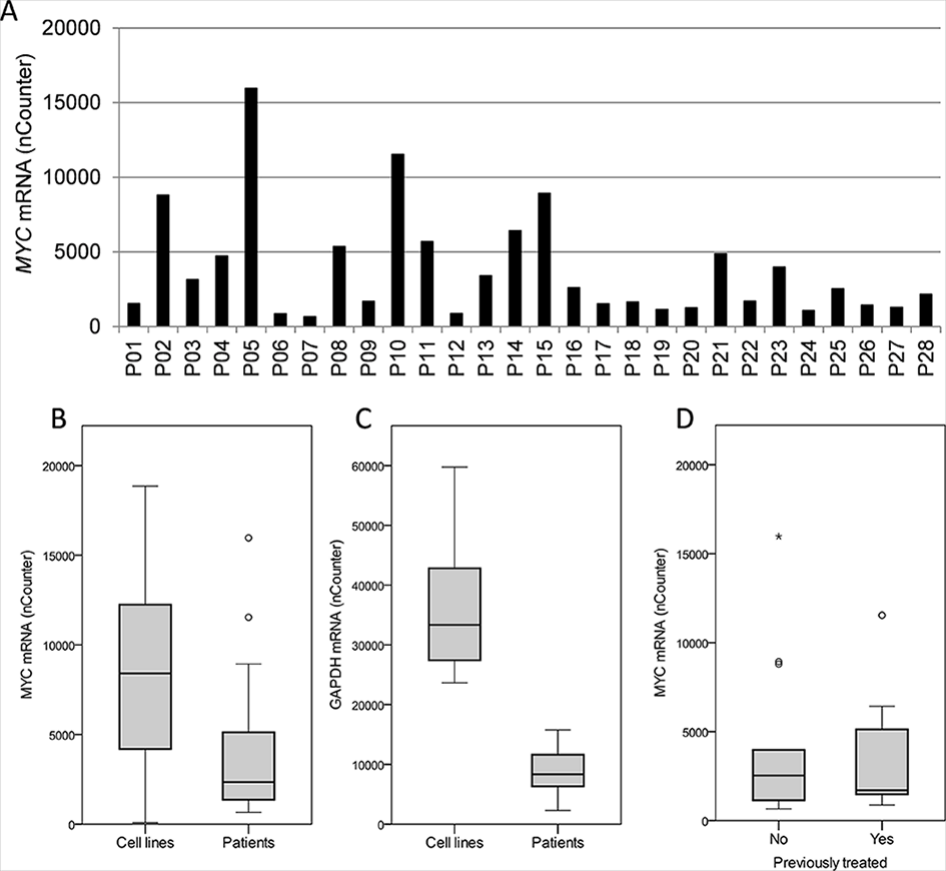
MYC and GAPDH mRNA levels in primary myeloma cells **A.** The levels of *MYC* mRNA in myeloma patient samples (*n* = 28), P01-P28, were measured using nCounter and the calculated mRNA copy numbers were plotted. Box plots comparing the levels of *MYC* **B.** and *GAPDH* **C.** mRNA in 28 isolated plasma cells from myeloma patients with 11 myeloma cell lines. **D.** The levels of *MYC* mRNA measured as in A were plotted for treated (*n* = 15) versus untreated (*n* = 13) patients in a box plot. The upper and lower borders of the box indicate upper and lower quartile, whereas the line inside the box indicates the median value. Whiskers indicate upper and lower adjacent values, whereas outside values are indicated by (o) and far out values are indicated by asterisks (*).

We went on to investigate the variation in *MYC* gene copy numbers in myeloma patient samples by the same method as applied for cell lines. Interestingly, most of the primary samples (*n* = 21) had two copies of the *MYC* gene and the samples deviating from this (*n* = 7) had *MYC* gene copies varying from 1 to 4 (data not shown). The levels of *MYC* mRNA, on the other hand, showed remarkable variation (Figure 3A). Thus, in contrast to myeloma cell lines, MYC levels in primary cells apparently are not determined by the number of gene copies as measured here, but by other mechanisms.

Interestingly, we originally compared *MYC* mRNA levels in cell lines and primary cells applying *GAPDH* mRNA as the only reference, getting higher MYC levels in primary cells than in myeloma cell lines. However, when comparing *GAPDH* mRNA levels in cell lines with primary cells using the Nanostring nCounter technology and using several genes as reference; it turned out that the difference in *GAPDH* mRNA was even greater than the difference in *MYC* mRNA. Thus, when comparing *MYC* mRNA levels in primary cells and cell lines, the patient cells as a group had lower *MYC* expression levels than cell lines (Figure 3B). Patients (*n* = 28) had a median *MYC* level of 2345, range 660–15969, whereas the cell lines (*N* = 11) had a median level of 8411, range 85–18859. Furthermore, the cell lines had a median *GAPDH* level of 33347, range 23653–59757, whereas the primary cells had a median *GAPDH* level of 8345, range 2297–15769 (Figure 3C). We also treated the primary myeloma samples with the MYC inhibitor to obtain IC50 values. However, the cell viability varied and only 18 out of 28 samples had a viability > 50% without treatment. Those samples were included in analysis (data not shown). When comparing IC50 values for 10058-F4 in the samples with baseline viability > 50% with *MYC* mRNA expression, we were not able to see the same correlation as in cell lines. However, analysis of only 18 samples may not be sufficient to see such correlations. There was no correlation between cell viability in the patient samples and 10058-F4 IC50 values (*n* = 28, data not shown).

The primary cells studied were both from newly diagnosed, untreated patients (*n* = 13), as well as from treated patients (*n* = 15), i.e. patients at a later stage of disease. Clinical characteristics of the patient samples are found in Supplementary Table I. Interestingly, the *MYC* mRNA levels were not different between previously treated and untreated patients (Figure 3D), indicating that untreated patients had myeloma cells with as high MYC expression as patients in later stages of disease. These observations suggest that both untreated and treated patients may have plasma cells with high MYC expression that possibly could be targeted by MYC inhibition.

## DISCUSSION

The most important data presented here is that there was a positive correlation between MYC expression and sensitivity to the MYC activity inhibitor, 10058-F4, in myeloma cell lines. That finding supports that MYC is important for myeloma cell survival and that the inhibitor specifically targets MYC. Secondly, although both myeloma cell lines and primary cells expressed high amounts of MYC, there are differences in the underlying mechanisms behind the expression. And thirdly, MYC expression was in the same range in plasma cells from untreated compared to treated patients indicating that MYC activation is an early oncogenic event in multiple myeloma.

Cell lines expressing the highest levels of MYC were the most sensitive to MYC-inhibition, indicating that these cells were more dependent on MYC for survival and proliferation than low-expressors. Thus, we hypothesize that patients with myeloma cells expressing high levels of MYC could benefit from MYC inhibition. MYC protein and mRNA are very unstable and may be rapidly degraded. When isolating primary myeloma cells from bone marrow samples the procedure usually takes a few hours and there is a risk that MYC protein and mRNA levels are not maintained at the same levels as when the cells are situated in the bone marrow. Thus, the most reliable way to measure MYC levels in patients could be by quantitative *in situ* immunohistochemistry using bone marrow biopsies. Unfortunately, it was not possible for us to perform such experiments in this study.

A major concern when applying small molecule inhibitors is the specificity of the inhibitor. The 10058-F4 drug has been proposed to be a specific inhibitor of MYC-MAX heterodimerization at concentrations < 100 μM. [20, 21] However, even if 10058-F4 clearly inhibits the MYC-MAX interaction, the compound may also affect other molecules within a cell. The correlation between MYC expression and sensitivity for the 10058-F4 drug found in cell lines indicated that the activity of the drug was specifically directed towards MYC activity. As MYC is a factor that is functionally non-redundant it may be particularly attractive as a therapeutic target. [22] MYC could already indirectly be targeted in the clinic since many drugs, such as dexamethasone and lenalidomide, have been shown to kill myeloma cells concomitantly with partial suppression of MYC, [23, 24] albeit, these drugs clearly also have other effects.

We found higher MYC mRNA levels in myeloma cell lines than in primary cells; although the range was approximately the same in these two groups. Nevertheless, gene copy number variations in *MYC* were only common in cell lines, indicating that primary cells had other ways of increasing MYC levels than cell lines. Indeed, recent publications indicate that different rearrangements involving *MYC* cause elevated MYC expression in nearly half of newly diagnosed patients. [13–15] MYC expression has been reported to affect cell proliferation and energy metabolism in rapidly proliferating cancer cells. [7] Amplification of the *MYC* gene seen in myeloma cell lines may be a consequence of the selection pressure for proliferation and survival in tissue culture flasks. Cell lines are highly proliferating, whereas primary myeloma cells in the bone marrow in general proliferate very slowly, with a Ki-67 labelling index less than 5% for most patients. [25] Amplification of the *MYC* gene may be a simple way of achieving high MYC levels in cell lines that over years have been selected for cell proliferation *in vitro*. Another difference is that, unlike myeloma cell lines, primary cells depend on the bone marrow microenvironment and are unable to survive in tissue culture. There was also a difference in *GAPDH* expression levels between cell lines and primary cells that could reflect differences in utilization of glycolysis for ATP production.

In patients, a negative relationship between MYC expression and progression-free and overall survival has been described, suggesting an important role for MYC in the regulation of tumor mass. [14, 26–28] We compared the MYC mRNA expression with clinical information on progression-free and overall survival in our patient samples, but could not find any relationship (data not shown). However, the number of informative patients was too low to draw any conclusions. Nevertheless, our findings that *MYC* mRNA is as high in untreated patients as in patients at later stages of disease suggest that MYC may be important during the whole course of disease.

To summarize, our results suggest that many myeloma patients, but first of all patients with cells expressing high MYC levels, might benefit from MYC inhibition. If a specific and clinically applicable MYC-inhibitor became available, it would be important to characterize which patients might benefit from inhibition of MYC.

## MATERIALS AND METHODS

### Cell lines and reagents

A panel of 11 myeloma cell lines was used. Four of the cell lines were in-house: OH-2, IH-1, URVIN and KJON, whereas 7 were from other sources: INA-6, CAG, JJN3 and ANBL-6 were kind gifts from Dr M. Gramatzki (University of Erlangen-Nurnberg, Erlangen, Germany), Dr J. Epstein (University of Arkansas for Medical Sciences, Little Rock, AR, USA), Dr J. Ball (University of Birmingham, UK), and Dr D. Jelinek (Mayo Clinic, Rochester, MN, USA), respectively, KMS-12-BM was obtained from DSMZ (Braunschweig Germany), and RPMI-8226 and U266 were from ATCC (Rockville, MD, USA). URVIN, INA-6 and ANBL-6 cells were grown in 10% heat inactivated fetal calf serum (FCS) in RPMI-1640 (RPMI) supplemented with interleukin (IL)-6 (1 ng/mL) (Biosource, Camarillo, CA, USA). CAG, JJN3, KMS-12-BM, RPMI-8226 and U266 were grown in RPMI with 10, 10, 20, 20 or 15% FCS, respectively. OH-2 and IH-1 were maintained in 10% heat-inactivated human serum (HS) (Department of Immunology and Transfusion Medicine, St. Olav's University Hospital, Trondheim, Norway) whereas KJON was maintained in 5% HS, all in RPMI and IL-6 (2 ng/mL). Cells were cultured at 37°C in a humidified atmosphere containing 5% CO_2_. For experiments 2% HS in RPMI was used as medium, with IL-6 (1 ng/mL) added for all IL-6 dependent cell lines.

### Primary cells

Primary CD138+ myeloma cells were isolated from bone marrow specimens included in the Norwegian Myeloma Biobank using RoboSep automated cell separator and Human CD138 Positive Selection Kit (StemCell Technologies, Grenoble, France). The study was approved by the Regional Ethics Committee (approval # 2011/2029) and all patients had given informed consent. Peripheral blood mononuclear cells (PBMC) were obtained from EDTA-blood from healthy controls by density gradient centrifugation using Lymphoprep (Axis-Shield, Oslo, Norway).

### Nucleic acid extraction

Genomic DNA and total RNA were extracted from frozen cell pellets using AllPrep DNA/RNA Micro Kit and Qiacube (Qiagen, Venlo, the Netherlands) following the manufacturer's instructions. The concentration and quality of DNA and RNA was determined using NanoDrop spectrophotometer (Thermo Fisher Scientific, Waltham, MA, USA), and samples were stored at −80°C until further use.

### Real-time quantitative reverse transcription PCR (qRT-PCR)

Complementary DNA (cDNA) was synthesized from total RNA using the High Capacity RNA-to-cDNA kit (Applied Biosystems, Carlsbad, CA, USA). PCR was performed using StepOne Real-Time PCR System and Taqman Gene Expression Assays (Applied Biosystems). The comparative Ct method was used to estimate relative changes in gene expression using MYC Taqman assay (Hs00153408_m1) and GAPDH (Hs99999905_m1) as housekeeping gene.

### Copy number variation (CNV)

Real-time reverse transcriptase quantitative polymerase chain reaction (real-time qPCR) was performed using the TaqMan Copy Number Assay Hs01764918_cn (MYC, exon 3), Hs00834648_cn (MYC, exon 2), Hs02758348_cn (MYC, exon 1), and TaqMan Copy Number Reference Assay (RNAse P) for internal control (Applied Biosystems). All PCR reactions were performed in triplets with genomic DNA using a StepOnePlus PCR system (Applied Biosystems). CNV was analyzed using CopyCaller Software (Applied Biosystems) and DNA isolated from PBMC was used for calibration.

### Gene expression analysis

For mRNA transcript counting the nCounter Human Cancer Reference Kit (cat.no GXA-CR1–12) and nCounter Technology (Nanostring Technologies, Seattle, WA, USA) was used. The standard mRNA Gene-expression experiment protocol provided by Nanostring was used, the only exception being that kit probes were diluted 1:2. Briefly, 100 ng total RNA from myeloma cell lines or patient samples was hybridized with reporter probes overnight at 65°C. The nSolver Analysis Software (Nanostring) was used for calculations of transcript numbers. Sample data was normalized against internal kit positive controls and the following housekeeping genes: *CLTC*, *GAPDH*, *GUSB*, *HPRT1*, *PGK1*, and *TUBB*.

### Immunoblotting

Cells were treated as indicated, washed with ice-cold phosphate-buffered saline (PBS) and lysed for 30′ on ice. The lysis buffer contained 1% NP40 (Sigma-Aldrich, St Louis, MO, USA), 150 mM NaCl, 50 mM Tris-HCl (pH 7.5), a protease inhibitor cocktail (Roche, Basel, Switzerland), 1 mM Na_3_VO_4_ and 50 mM NaF. Samples were electrophoresed on pre-cast agarose gels and blotted onto nitrocellulose membranes using the NuPAGE system (Life Technologies, Carlsbad, CA, USA). The membranes were blocked with 5% nonfat dry milk in Tris-buffered saline with 0.01% Tween 20 (TBS-T). The primary antibodies used were c-MYC (RRID: AB_2148606, Cat# 551102, BD Biosciences, Trondheim, Norway), and GAPDH (RRID:AB_2107448, Cat# Ab8245, Abcam, Cambridge, UK). The secondary antibody was horseradish peroxidase-conjugated goat-anti-mouse (Dako Cytomation, Glostrup, Denmark). Positive bands were detected using the luminescence substrate SuperSignal West Femto (Thermo Fisher Scientific) and Odyssey Fc imager (LI-COR Biosciences, Ltd., Cambridge, UK).

### Cell proliferation assay

Cell proliferation was estimated using the CellTiter-Glo assay (Promega, Madison, WI, USA), that measures the cells' ATP content. The cells were seeded in 100 μL per well in white opaque 96 well plates and treated with increasing doses of inhibitor for 72 hours. The cell lines have different growth rates and, thus, the cell numbers that were seeded for each cell line varied from 10000 – 50000 per well. Assay reagent was added and the plates were mixed for 2 min on a shaker. After a 10 min incubation in room temperature luminescence was detected using a Victor 1420 multilabel counter (PerkinElmer Inc., Waltham, MA, USA).

### Statistical analysis

Statistical analysis and box plots were made in IBM SPSS Statistics 20 (IBM Corp., Armonk, NY, USA).

## SUPPLEMENTARY DATA: TABLE AND FIGURES


