# Correction: *MYC* amplifications in myeloma cell lines: correlation with MYC-inhibitor efficacy

**DOI:** 10.18632/oncotarget.26373

**Published:** 2018-11-13

**Authors:** Toril Holien, Kristine Misund, Oddrun Elise Olsen, Katarzyna Anna Baranowska, Glenn Buene, Magne Børset, Anders Waage, Anders Sundan

**Affiliations:** ^1^ K.G. Jebsen Center for Myeloma Research, Department of Cancer Research and Molecular Medicine, Norwegian University of Science and Technology, Trondheim, Norway; ^2^ Department of Immunology and Transfusion Medicine, St. Olav’s University Hospital, Trondheim, Norway; ^3^ Department of Hematology, St. Olav’s University Hospital, Trondheim, Norway; ^4^ CEMIR (Centre of Molecular Inflammation Research), Department of Cancer Research and Molecular Medicine, Norwegian University of Science and Technology, Trondheim, Norway

**This article has been corrected:** The legends for figures 1 and 2 were accidentally switched. The corrected figure legends are given below:

**Figure 1: Expression of MYC in myeloma cell lines correlated positively with sensitivity to MYC inhibition.** The IC50-values of the MYC inhibitor 10058-F4 calculated from the results shown in Supplementary Figure 2 was compared with **A.** *MYC* mRNA values or **B.** MYC/GAPDH relative protein levels. The slope and R^2^-values are shown in the plots.

**Figure 2: *MYC* gene copy numbers determine expression of MYC mRNA and protein in myeloma cell lines.** In a panel of myeloma cell lines the levels of *MYC* gene copy numbers as measured by PCR was related to **A.** *MYC* mRNA measured using nCounter, and **B.** MYC protein levels measured using immunoblotting and normalized to GAPDH. The slope and R^2^-values are shown in the plots.

Original article: Oncotarget. 2015; 6:22698-22705. https://doi.org/10.18632/oncotarget.4245

