# Supplementary material for: MYC amplifications in myeloma cell lines: correlation with MYC-inhibitor efficacy
Source: Oncotarget. 2015 Jun 2;6(26):22698–705. doi: 10.18632/oncotarget.4245 (PMC4673192; doi:10.18632/oncotarget.4245)
Supplement: Supplementary file 1 [file oncotarget-06-22698-s001.pdf]

SUPPLEMENTARY DATA

Holien T., *et. al.*, MYC amplifications in myeloma cell lines; correlation with MYC-inhibitor efficacy.

Supplementary Table S1. Patient characteristics

Patient number	Gender and age	Treated	CD138 + cells in BM (%)	ISS-stage	WHO performance status	Secretory Ig-class	t(4;14)	del17
P01	M-73	Yes	95	ND	3	IgA kappa	No	No
P02	F-83	No	87	2	1	non-secretory	Yes	No
P03	M-69	No	60	ND	2	IgG kappa	No	Yes
P04	M-69	Yes	98	ND	3	IgG lambda	No	No
P05	F-71	No	20	1	1	IgA kappa	Yes	No
P06	F-73	No	24	1	1	lambda	No	No
P07	F-69	No	65	2	2	IgA lambda	No	No
P08	F-67	Yes	90	ND	2	IgG lambda	No	Yes
P09	F-67	Yes	100	ND	3	IgG lambda	No	Yes
P10	F-61	Yes	19	ND	3	kappa	No	No
P11	F-77	Yes	36	ND	3	IgG lambda	No	No
P12	M-75	Yes	4	ND	2	IgA kappa	No	No
P13	M-72	Yes	10	2	0	lambda	ND	ND
P14	F-51	Yes	20	1	1	IgA kappa	No	Yes
P15	F-72	No	50	1	1	IgA kappa	Yes	No
P16	F-60	No	25	1	1	IgA lambda	No	No
P17	F-69	Yes	95	3	0	IgA kappa	No	No
P18	M-69	No	23	1	1	IgG kappa	No	No
P19	F-84	No	60	3	0	IgA kappa	Yes	No
P20	F-50	Yes	58	ND	1	lambda	No	Yes
P21	M-70	Yes	28	ND	2	IgG lambda	Yes	No
P22	F-78	Yes	33	ND	1	IgA kappa	Yes	No
P23	F-82	No	50	3	0	IgG kappa	No	No
P24	M-72	No	40	1	0	IgA lambda	No	Yes
P25	M-60	No	80	3	ND	IgG	No	No
P26	F-78	Yes	42	ND	1	IgA kappa	Yes	No
P27	F-58	Yes	80	ND	2	IgG kappa	No	No
P28	M-36	No	90	2	2	kappa	No	No

Abbreviations: BM, bone marrow; ISS, international staging system; ND, not determined; WHO, World Health Organization.

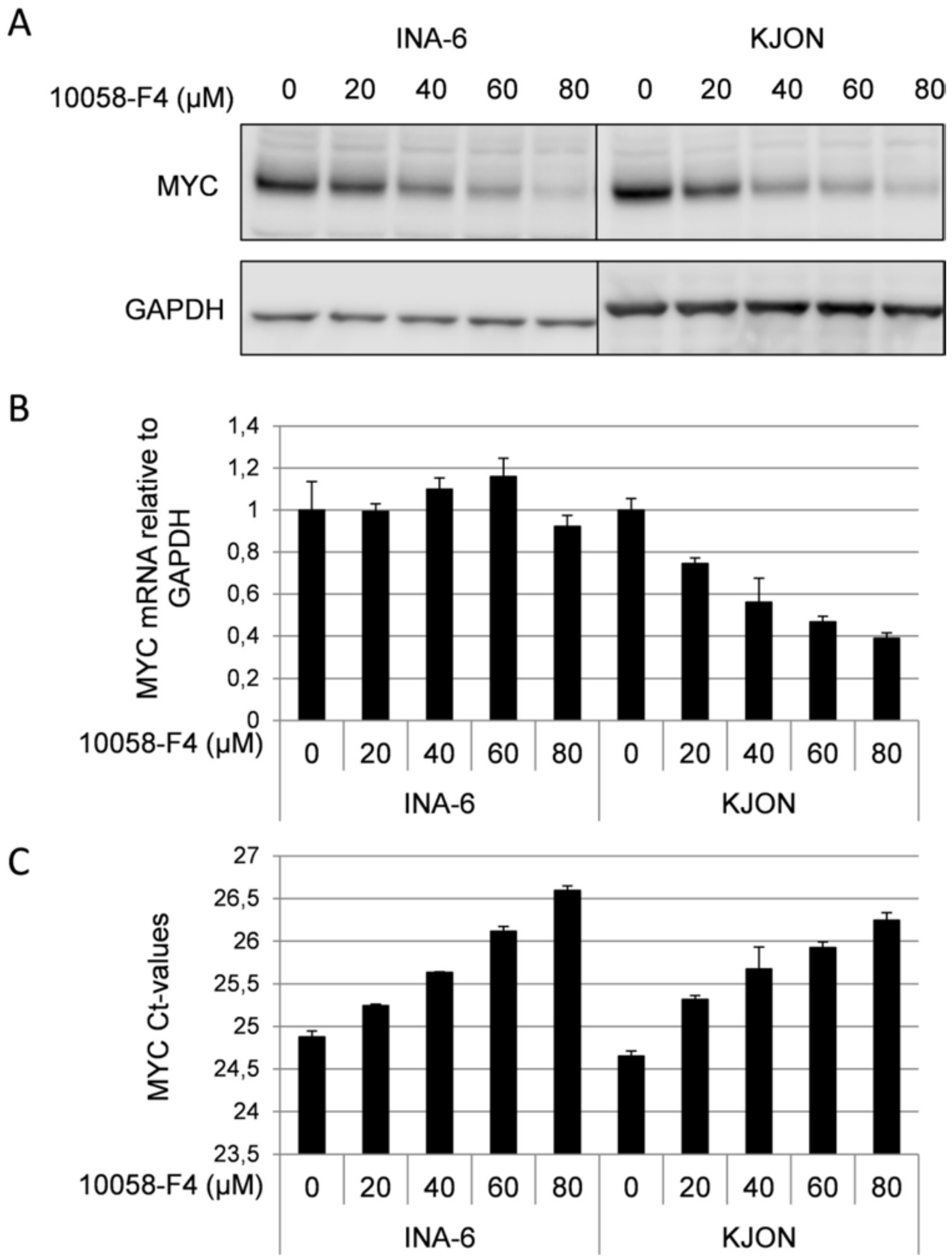

Supplementary Figure S1: Dose dependent effects of 10058-F4 on MYC protein and mRNA levels. INA-6 and KJON myeloma cell lines were treated with increasing concentrations of 10058-F4 for 6 hours and cells were subjected to **A.** immunoblotting with MYC and GAPDH antibodies, and **B.** QRT-PCR of MYC mRNA where the relative mRNA levels was calculated using the delta delta Ct method with GAPDH as housekeeping gene. **C.** represents the Ct-values for the MYC gene from (B) before normalization**.**

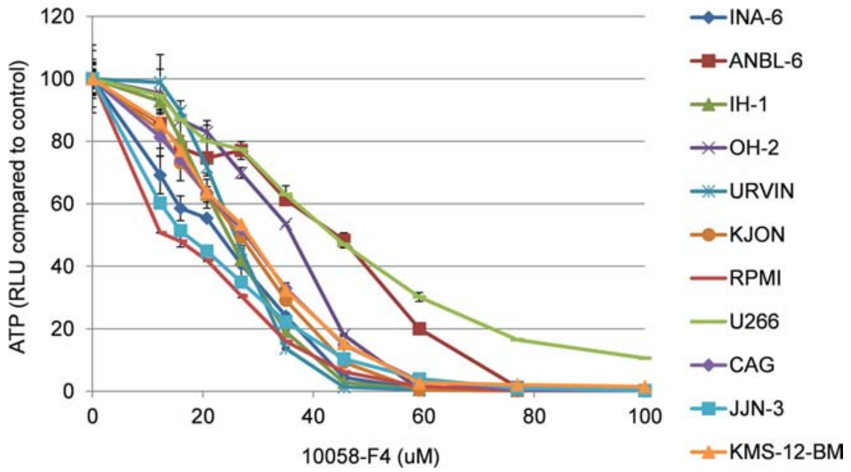

Supplementary Figure S2: Effects of MYC inhibition on growth of myeloma cell lines. Cells were seeded in in 96 well plates in 2% HS in RPMI with addition of IL-6 (1 ng/mL) for IL-6 dependent cell lines. Increasing concentrations of the 10058-F4 MYC-inhibitor was added. The plates were incubated for three days before addition of CellTiter Glo substrate and the luminescence was read on a luminometer. The light intensity correlates with ATP levels as a measure of number of viable cells in the wells. The relative luciferase units were plotted and related to untreated control. The graph shows a representative experiment of at least three for each cell line. Error bars indicate +/− 1 SD of triplicate measurements.

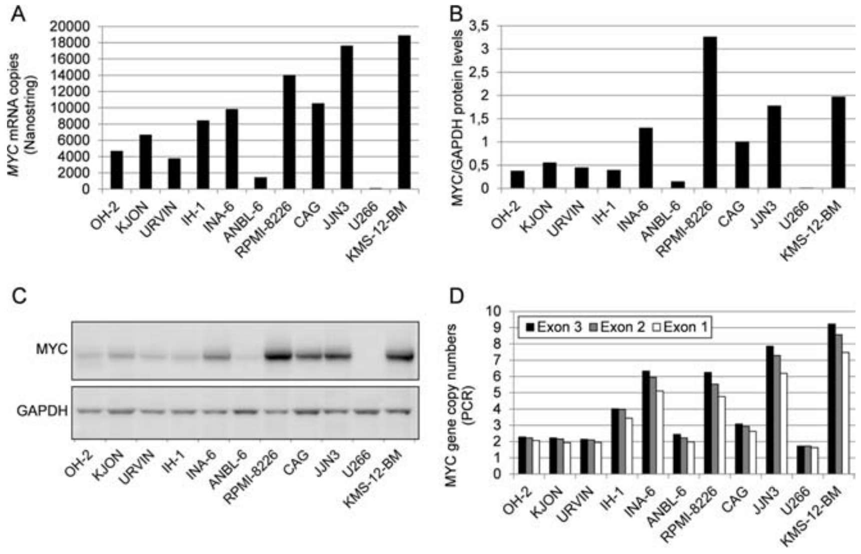

Supplementary Figure S3: MYC gene copies, mRNA and protein levels in a panel of multiple myeloma cell lines. **A.** Total RNA was isolated and used for MYC mRNA transcript measurements with nCounter. **B.** Protein levels of MYC in cell lines normalized to GAPDH levels based on immunoblotting **C. D.** Real-time qPCR-based *MYC* copy number analysis was performed using genomic DNA. Three probes were used, each detecting different exons as indicated.
